# A Novel Method to Simulate the Progression of Collagen Degeneration of Cartilage in the Knee: Data from the Osteoarthritis Initiative

**DOI:** 10.1038/srep21415

**Published:** 2016-02-24

**Authors:** Mika E. Mononen, Petri Tanska, Hanna Isaksson, Rami K. Korhonen

**Affiliations:** 1 Department of Applied Physics, University of Eastern Finland, Kuopio, Finland; 2 Department of Radiology, Oulu University Hospital, Oulu, Finland; 3 Department of Biomedical Engineering, Lund University, Lund, Sweden; 4 Diagnostic Imaging Centre, Kuopio University Hospital, Kuopio, Finland

## Abstract

We present a novel algorithm combined with computational modeling to simulate the development of knee osteoarthritis. The degeneration algorithm was based on excessive and cumulatively accumulated stresses within knee joint cartilage during physiological gait loading. In the algorithm, the collagen network stiffness of cartilage was reduced iteratively if excessive maximum principal stresses were observed. The developed algorithm was tested and validated against experimental baseline and 4-year follow-up Kellgren-Lawrence grades, indicating different levels of cartilage degeneration at the tibiofemoral contact region. Test groups consisted of normal weight and obese subjects with the same gender and similar age and height without osteoarthritic changes. The algorithm accurately simulated cartilage degeneration as compared to the Kellgren-Lawrence findings in the subject group with excess weight, while the healthy subject group’s joint remained intact. Furthermore, the developed algorithm followed the experimentally found trend of cartilage degeneration in the obese group (R^2^ = 0.95, p < 0.05; experiments vs. model), in which the rapid degeneration immediately after initiation of osteoarthritis (0–2 years, p < 0.001) was followed by a slow or negligible degeneration (2–4 years, p > 0.05). The proposed algorithm revealed a great potential to objectively simulate the progression of knee osteoarthritis.

Abnormal loading of the knee joint, *e.g.* due to a joint disorder or overweight, may cause excessive forces in specific regions of the joint surfaces and alterations in tissue responses, likely leading to the development of osteoarthritis^1^,^2^,^3^. At present, osteoarthritis cannot be prevented effectively. The total cost of osteoarthritis has been estimated to be between 1% and 2.5% of the total gross domestic product in western countries^4^. In Europe, over 100 million people have arthritis, and in the United States, the direct costs of arthritis were $51.1 billion in 2004^5^. As the number of elderly people increases, osteoarthritis represents a major economic burden on the health care systems and is a serious threat to the quality of life within the populations. For instance, in the UK in 2010, there were 4.7 million doctor appointments due to knee osteoarthritis for patients over 45 years. It has been predicted that this number will increase to 6.7 million by 2051^6^. The most cost-efficient and helpful approach for the disease would be prevention. However, this is currently not possible. Rather, the disease typically develops slowly until it reaches the stage where the only solution is total joint replacement. This entire process is painful and expensive. In order to prevent osteoarthritis effectively, there should be a quantitative estimate (*e.g.*, via an adaptive algorithm) that could simulate the development of osteoarthritis in a patient-specific manner. This algorithm should be able to guide patients towards the best possible intervention (*e.g.*, weight loss, surgery, rehabilitation) to prevent or delay the onset and/or progression of osteoarthritis.

A framework for the initiation and progression of knee osteoarthritis was originally presented by Andriacchi *et al.*^7^. Briefly, the initiation phase of knee osteoarthritis is controlled by abnormal joint kinematics and excessive loading, which cause damage to the collagen fibril network. During the progression phase, the rate of knee osteoarthritis progression is strongly controlled by the weight of the subject. It has also been suggested that not only excessive, impact loading, but also loading duration causes a risk for osteoarthritis^8^. Interestingly, even though adaptive cartilage models that can simulate tissue growth and development exist^9^,^10^,^11^,^12^,^13^, there have not been any attempts to implement these or other concepts to simulate cartilage degeneration during the progression of osteoarthritis.

Obesity is one of the most significant risk factors for the onset and development of knee osteoarthritis, largely due to excessive chronic joint loads^2^,^3^. Based on the baseline data extracted from 1087 patients in the osteoarthritis initiative database (OAI – http://www.oai.ucsf.edu/), body mass index (BMI) is directly linked to the development of osteoarthritis (Fig. 1). Randomized controlled trial studies have also shown statistically the effect of overweight and interventions on the progression of osteoarthritis in a group level^14^,^15^,^16^. However, even for overweight or obese subjects, it is currently not possible to show quantitatively to what extent osteoarthritis of a subject develops if it develops at all.

In the present study, we developed a cartilage degeneration algorithm aiming to simulate the development of osteoarthritis in the knee for overweight subjects. The algorithm was based on cartilage overloading so that cumulatively accumulated excessive stresses (above failure limit) caused alterations in tissue properties with time. For computational simulations, finite element modeling was applied with information of the knee joint cartilage obtained from MRI. The simulation results were validated against Kellgren-Lawrence grades obtained from x-ray data of the same subject groups (baseline and 4-year follow-up, OAI – http://www.oai.ucsf.edu/). The presented algorithm provides a novel approach for subject specific simulation of the development of knee osteoarthritis and cartilage degeneration due to excessive chronic knee joint loading.

## Results

### Osteoarthritis Initiative subjects

**Figure 1:**
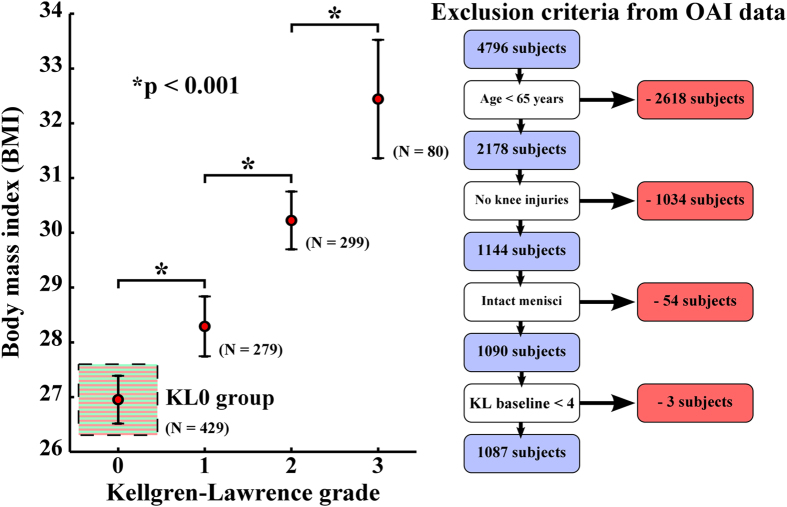
Relationship between body mass index and joint degenerative (Kellgren-Lawrence) grade (mean ± 95% CI) based on the baseline data of 1087 subjects from osteoarthritis initiative (OAI – http://www.oai.ucsf.edu/) database. Specific exclusion criteria for 1087 subjects are presented on the right. Subjects with initially zero Kellgren-Lawrence grade at the baseline are highlighted using green and red stripes (these subjects were further divided into normal weight and obese groups, Fig. 2). Statistically significant differences between adjacent Kellgren-Lawrence grading groups are indicated with a black star (*p < 0.001, one-way ANOVA with Tukey’s Post-hoc test).

**Figure 2:**
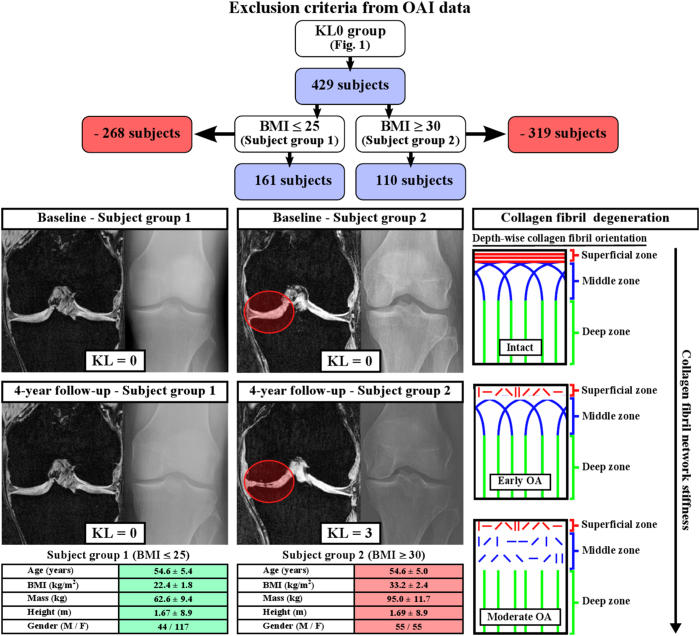
Information of subjects group 1 (first column) and 2 (second column) with baseline and 4-year follow-up MRI and x-ray data (Kellgren-Lawrence grades) of representative subjects and schematic presentation of the collagen fibril degeneration in osteoarthritis (third column). In our algorithm, cumulative accumulated excessive loading leads to collagen damage and tissue degeneration, leading to reduced stiffness of the collagen fibril network^61^. Specific exclusion criteria for subject groups 1 (BMI 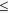 25) and 2 (BMI 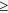 30), obtained from the KL0 group in Fig. 1, are presented on top.

Information from two subject groups out of 429 patients (initially Kellgren-Lawrence grade = 0, Fig. 1: KL0 group) with ages below 65 years were evaluated from OAI database consisting of specific information of 4796 subjects (Figs 1 and 2); a group without a risk for osteoarthritis (BMI 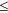 25 – no meniscus injuries – never operated meniscus or cartilage, no knee injuries – never injured badly enough to limit the ability to walk for at least two days) and a group with a high risk for osteoarthritis due to overweight (BMI 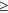 30 without meniscus and knee injuries). Representative subjects from both subject groups were then selected for testing and validating the degeneration algorithm; normal weight (BMI = 24, without osteoarthritic changes) and obese (BMI = 35, with osteoarthritic changes) test subjects of the same gender and of similar age and height (Fig. 2, see specific subject group details here).

### Knee joint model and degeneration algorithm

**Figure 3:**
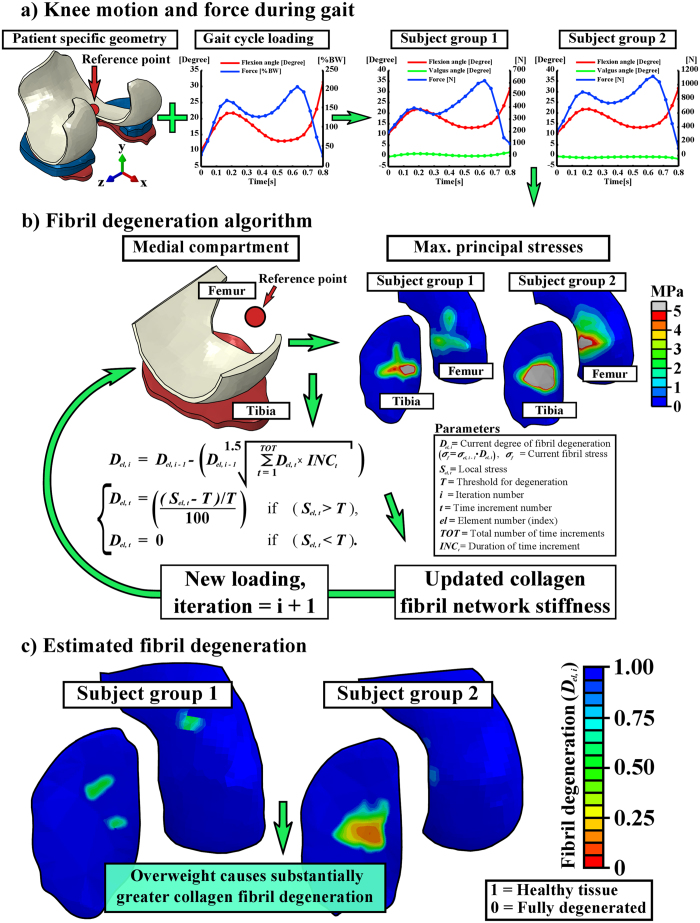
Workflow from the subject-specific knee geometry with gait loading (**a**) to the fibril degeneration algorithm (**b**) and the results after iterative degenerative simulations (**c**). (a-left) Knee joint loads obtained from the literature^24^,^28^ were implemented into the subject-specific whole knee joint models. (a-right) The outcomes (force and rotations) of the whole knee joint model were implemented into the medial compartment model. (**b**) The fibril degeneration algorithm (left), based on excessive stresses (right), was applied into the medial compartment of the knee joint (most of the experimentally observed degeneration occurred there). The shown stress distributions are from the first peak loading force of the stance phase of gait. (**c**) Subject-specific fibril degenerations for both subject groups (healthy and obese). Used joint forces and rotations were considered as relative motions of femur with respect to tibia and they were implemented into a reference point located at the middle-central point between the medial and lateral epicondyles of the femur (**a**) (see details from Methods). In medial compartment models, (**b**) the location of the reference point was identical with that in the whole knee joint models (**a**).

The subject specific geometries of the right knee joints were first segmented from the MRI data (Figs 2 and 3a). These were then implemented, together with gait cycle loading (walking), into the computational finite element models. After the whole knee joint models were simulated, the medial compartment models with a cartilage degeneration algorithm were constructed and collagen fibril degenerations within subject groups 1 and 2 were simulated (Fig. 3a–c) (see details and justifications from Methods). Articular cartilage in the models was considered as a fibril reinforced poroviscoelastic material, including all major tissue constituents in cartilage, *i.e.*, collagen fibrils, proteoglycans and fluid (see details from Methods and Table 1). Menisci were modeled as a transverse isotropic material.

**Figure 4:**
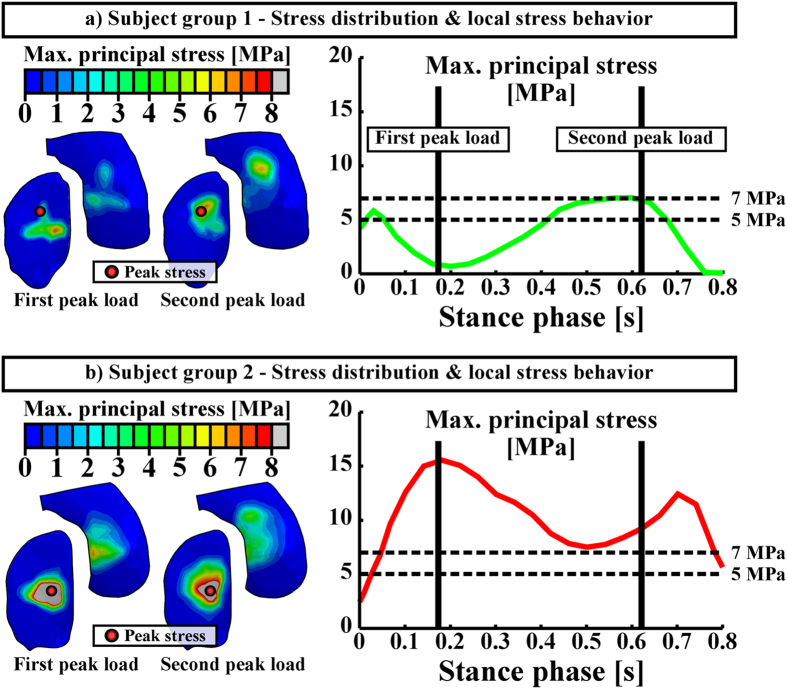
Maximum principal stresses in the subject group 1 (**a**) and 2 (**b**) models. Left column presents stress distributions on the tibial cartilage surface during the first and the second peak loading forces (loading response and terminal extension, respectively), whereas right column presents peak maximum principal stresses during the stance phase of gait. Vertical solid lines in the right column indicate the first and the second peak loading forces, while horizontal dashed lines indicate different threshold levels for collagen fibril degeneration.

Since one of the first signs of osteoarthritis is collagen fibrillation^1^, extensive collagen damage is considered to be irreversible^17^, and collagen is known to strongly control the mechanical response of cartilage under dynamic loading such as walking^18^,^19^,^20^, only collagen was allowed to degrade in the algorithm (Fig. 2, right). In the adaptive collagen fibril degeneration algorithm, tensile tissue stresses, *i.e.*, maximum principal stresses (controlled by the collagen fibrils), were used to control the rate of degeneration during each iterative simulation step (Fig. 3b). The collagen fibril damage (and subsequent degeneration^21^) occurred at the cartilage regions where the tensile tissue stress exceeded a threshold limit of 5 MPa. This failure limit is in accordance with a previous experimental study^22^. Maximum principal stresses (tensile stresses) in the subject group 2 (obese) were substantially higher than those in the subject group 1 (normal weight), and they exceeded the 5 MPa threshold failure limit almost during the entire gait cycle for the obese subject group (Fig. 4). Thus, duration of the loading in different regions (or repetitive loading) was also needed to control the degeneration (see more below)^8^.

In the degeneration algorithm, the stress of the collagen fibril network was altered locally according to the chosen criteria (see Fig. 3b and details from Methods). The current degree of fibril degeneration 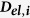 in each element 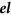 was calculated by the weighted sum after each gait cycle 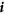 as follows:


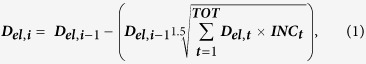


where 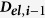 is the degree of fibril degeneration in each element 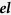 after the previous gait cycle 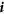 (when 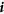 = 1 → 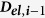
** **=** **1), 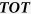 is the total number of time increments used to represent each gait cycle simulation, 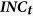 is the duration of each time increment 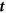, and 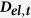 is the calculated fibril degeneration factor for an individual element 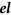 after each time increment 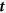. This degree of fibril degeneration altered then directly the fibril stress (Fig. 3b), with more degenerated tissue having softer collagen fibril network (Fig. 3c). In this algorithm, the duration of each time increment 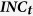 was used as a weight for each fibril degeneration factor calculated during each time increment. As a result of this assumption, the collagen fibril degeneration was higher at locations where the tissue experienced more cumulatively accumulated loading (where excessive stresses were observed more frequently, see equation 1). Similar cartilage degeneration mechanism has been proposed in a review by Seedhom^8^. The fibril degeneration factor in equation (1) was calculated with the local stress 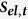 during each time increment in each individual element 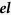 and with the constant threshold limit for fibril degeneration 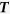 as follows (Fig. 3b):


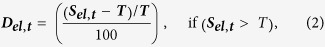



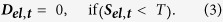


In biophysical terms, the calculated fibril degeneration factor (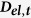) describes the amount of fibril degeneration at each element as a function of time (phase of stance), where 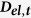
** **=** 0** means no degeneration and 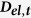
** **=** 1** means fully degenerated collagen fibrils (See equation (1)).

**Figure 5:**
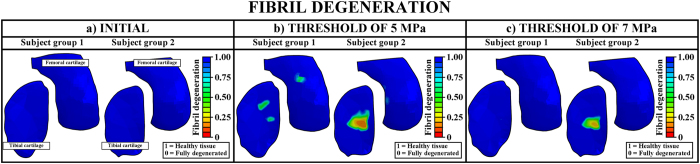
Collagen fibril degeneration distributions in (**a**) baseline (before the degeneration algorithm is applied) and at the end of the simulations with the thresholds of (**b**) 5 MPa and (**c**) 7 MPa.

**Figure 6:**
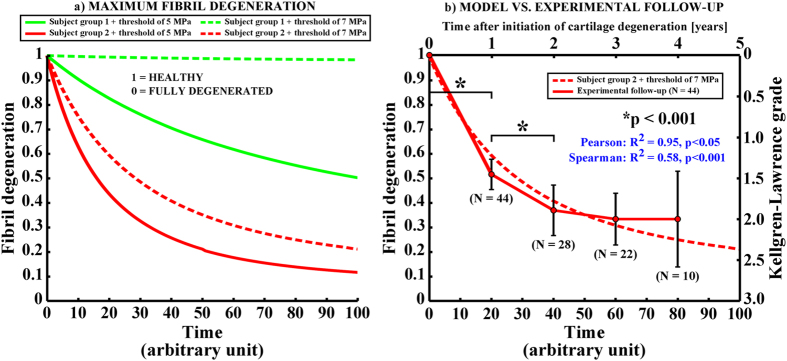
(**a**) The maximum fibril degeneration as a function of time (arbitrary units) with thresholds of 5 MPa (solid line) and 7 MPa (dashed line) in the subject group 1 (normal weight, green) and 2 (obese, red). The curves are obtained at the location where the highest fibril degenerations occurred (see locations from Fig. 4), when using the threshold of 5 MPa for the collagen fibril degeneration. (**b**) The fibril degeneration in the subject group 2 with the threshold of 7 MPa along with an experimentally observed increase in Kellgren-Lawrence grade during the follow-up period for 44 knee joints from 35 obese test subjects (mean ± 95% CI, see also Fig. 2). Since for many patients cartilage degeneration was not initiated during the first year of the follow-up period, the number of patients (N) decreases as a function of time. Nonetheless, the same trend is seen in tissue degeneration both in our novel degeneration algorithm and in experiments. Statistically significant differences in Kellgren-Lawrence grades between adjacent time points are indicated with black stars (*p < 0.001, one-way ANOVA with Tukey’s Post-hoc test). Correlations between experimental follow-up data and simulated cartilage degeneration were calculated with Pearson and Spearman tests using mean experimental values and all observations, respectively.

In order to simulate the model in a reasonable time (which is important in clinical practice), we used 100 iterations as the total simulation time (in arbitrary units). The simulations could also be adjusted into real time (4 years). However, at the moment that is not reasonable due to the computation time. After the iterative degeneration simulations with a threshold of 5 MPa tensile stress, substantial differences in the collagen fibril degeneration between subject groups 1 and 2 were observed (Figs 3c, 5 and 6). In the subject group 1, only minor collagen fibril degeneration was simulated in two small regions in the tibial cartilage at the tibiofemoral load-bearing area. In the subject group 2, the maximum degree of collagen fibril degeneration in the tibial cartilage was three times higher than that in the subject group 1. The current results were consistent with experimental baseline and 4-year follow-up data (Figs 2 and 6).

As indicated above, the threshold limit of 5 MPa for the initiation and development of the collagen fibril degeneration was chosen based on the experimentally measured failure stress of cartilage^22^, evaluated with a strain rate of 5 mm/min. However, during impact loading (*e.g.*, during gait), the strain rate can reach up to 10 mm/s^23^,^24^. This may influence the threshold limits^25^. There are also other factors such as gender, age, and site (femur and tibia) which may affect the threshold^26^,^27^. Therefore, we also tested the adaptive algorithm with different threshold limits. With a threshold limit of 7 MPa, no fibril degeneration was simulated for the subject group 1 (healthy), while high degree of fibril degeneration was still simulated for the subject group 2 (obese) (Figs 5 and 6).

### Validity of the models

Obviously, higher degree of collagen fibril degeneration was estimated with the lower threshold value (5 MPa) for the collagen fibril degeneration. By using the threshold of 5 MPa for the fibril degeneration, the maximum local collagen fibril degenerations in one element were up to ~50% and ~88% in subject groups 1 and 2, respectively (with a scale of 0–100%). When the threshold of 7 MPa was implemented, the maximum fibril degenerations were up to ~2% and ~79% in subject groups 1 and 2, respectively (Figs 5 and 6a). Since the radiographic grade of osteoarthritis remained unchanged in the subject group 1 during the entire follow-up period (4 years), while osteoarthritis had developed severely in the subject group 2 (as indicated by the significantly increased Kellgren Lawrence grades, Fig. 6b), for these test subject groups, the threshold of 7 MPa for the collagen fibril degeneration is considered more realistic (Figs 2, 5 and 6).

In the models, the collagen fibril degeneration progressed nonlinearly and the highest degenerations were observed at the initial stages of osteoarthritis (Fig. 6a). This is consistent with the experimental results with obese subjects obtained from the osteoarthritis initiative (OAI – http://www.oai.ucsf.edu/) database (Figs 2 and 6b). Correlations between experimentally observed Kellgren-Lawrence grades during the progression of osteoarthritis and simulated fibril degenerations were significant (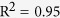, p < 0.05, Pearson for mean values of Kellgren-Lawrence grades and 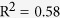, p < 0.001, Spearman for all observations). Within this subject group (Fig. 2), those who experienced an increase in the Kellgren-Lawrence grade from zero to higher levels (baseline Kellgren-Lawrence grade = 0) at some point (not necessarily from 0 to 1 year) during the 4-year follow-up, the Kellgren-Lawrence grade increased significantly during first two years (*N* = 44, p < 0.001, Fig. 6b). Following this, cartilage degeneration decelerated (p > 0.05 between Kellgren-Lawrence grades from 2 to 4 years after initiation of osteoarthritis) and showed similarly nonlinear behavior in cartilage degeneration with the degeneration algorithm. It should be noted that experimentally observed progression of osteoarthritis occurred in 35 out of 110 obese (BMI 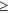 30) subjects (Figs 2 and 6). Similar nonlinear Kellgren-Lawrence behavior as a function of time occurred also among subjects with knee injuries and the same age range (data from the osteoarthritis initiative, N = 30, mean age = 56 years, mean BMI = 28, results not shown). These findings are also perfectly consistent with a previous experimental study^27^, in which a subject group with knee pain and an average age of 45 years was followed for 12 years. In that study, the highest increase in the Kellgren-Lawrence grade was observed during the first 5 years, while only minor changes were observed between 5-year and 12-year follow-ups.

## Discussion

A cartilage degeneration algorithm was implemented into the human knee joint for the first time. The algorithm was based on the fact that cartilage tissue is vulnerable to cumulatively accumulated excessive tensile stresses of collagen due to excessive joint loads. The developed computer aided approach revealed a great potential to simulate the development of osteoarthritis in a subject-specific manner within the knee joints based solely on the information obtained from MRI. The simulated onset and development of osteoarthritis agreed with experimental baseline and 4-year follow-up data, supporting the validity of our method. The presented work provides therefore an important and groundbreaking step toward developing a rapid and subject-specific diagnostic tool for the simulations of the onset and development of knee osteoarthritis and cartilage degeneration related to excessive chronic loading due to overweight.

Osteoarthritis is a whole organ disease affecting both cartilage and bone. Early signs of osteoarthritis in cartilage are collagen fibrillation (causing collagen fibril network softening), increase in fluid fraction and proteoglygan depletion. At later stages of osteoarthritis, aforementioned alterations are amplified and cartilage loss (thinning) initiates^1^. Kellgren-Lawrence grade is not a linear scale and it is a composition of bone and cartilage changes so that level 1 describes doubtful joint space narrowing and possible osteophytic lipping, level 2 describes possible joint space narrowing and define osteophytes, level 3 describes definite joint space narrowing and multiple osteophytes, and level 4 describes marked joint space narrowing and large osteophytes. In our degeneration algorithm, we did not take all aforementioned osteoarthritic changes into account but focused only on the collagen fibril network degeneration of cartilage, since the damage in collagen is virtually irreversible^17^. Furthermore, our algorithm was based on mechanical overloading and it has been shown that the collagen fibril network stiffness controls cartilage strains and stresses during walking^19^. Though cartilage loss was not considered in the algorithm, bone surfaces became closer to each other during joint loading when cartilage became softer during progression of osteoarthritis, similarly as would happen with cartilage loss.

Even though anatomical factors and alignment were obtained from MRI and included in the models, experimental data from the gait loading was not subject specific and was based on literature data from instrumented *in vivo* tibial implants^24^,^28^. This can be considered as a limitation and may be the reason why in the obese subject the degeneration location was simulated ~5 mm more lateral compared to the 4-year follow-up MRI data (Figs 2 and 5). However, it should be noted that in the current approach, the subject-specific gait data is an additional option (see Methods). The subject-specific movement data might improve the accuracy of the simulation. However, in clinical use the method presented here must be rapid and based on available data. If patients with a sport injury, joint disorder or pain due to osteoarthritis go to medical examination and imaging in special healthcare, this typically provides MRI data, whereas obtaining subject-specific gait data is not feasible. Therefore, it is actually strength of the model that it is able to simulate the development of osteoarthritis based only on the imaging information.

Though knee osteoarthritis may be initiated also from the patellofemoral compartment or lateral tibiofemoral compartment^29^, our cartilage degeneration algorithm for knee osteoarthritis was validated only for the medial tibiofemoral compartment. In previous studies^30^,^31^,^32^, it has been reported that osteoarthritic changes are more frequent in the medial compartment compared to the lateral compartment, especially within obese subjects. Thus our approach to use only the medial compartment is justified. We did not have patella, ligaments or muscle forces in the model. However, the effects of these are already considered in the knee joint forces of the computational model. Thus, their presence in the model was not necessary. The method presented here is capable of taking into account all compartments, but using such geometry with the iterative degeneration algorithm would require a lot more computational time. Simulating one compartment while considering the whole knee joint motion, as was done here, allows the use of denser FE meshes in the whole knee joint model, which also reduces the computational time. Nonetheless, the lack of the patellofemoral joint limits the use of our current model to the tibiofemoral joint compartment.

Used test subjects were selected based on the baseline and 4-year follow-up Kellgren-Lawrence grades. Kellgren-Lawrence indicates joint space width between femur and tibia based on x-ray and is a rough estimate of osteoarthritis. MRI scoring (WORMS) is more sensitive compared to Kellgren-Lawrence grading to identify subjects with early osteoarthritis^33^. However, in the current study, we concentrated on studying moderate tibiofemoral cartilage degeneration and Kellgren-Lawrence grading was therefore adequate. We also used 4-year follow-up MRI data to locate the degenerated areas which were then compared to the results obtained from the degeneration algorithm (see above).

It has been suggested in many studies that excessive and cumulative loadings are risk factors for knee osteoarthritis^8^,^34^,^35^,^36^. These two factors were accounted for in the presented degeneration algorithm (as well as anatomical factors). Changes in physical activity levels were not considered in the presented algorithm. The relationship between physical activity and the onset of knee osteoarthritis has been studied in humans^35^,^37^ and by using animal models^38^. Based on these studies^37^,^38^, physical activity levels do not correlate linearly with the onset of osteoarthritis and it has been suggested that cartilage tissue remains “healthier” when physical activity levels are moderate compared to high intensity/endurance physical activity or immobilization. From the mechanical point of view, the relationship between physical activity and osteoarthritis is the same for normal weight and overweight subjects, and different tissue condition (normal, degenerated) may be for instance due to different interstitial fluid flow and pressure, nutrient transport and cell responses as a result of different mechanical forces experienced by the tissue and cells^14^,^38^,^39^. In addition to physical activity levels, our algorithm did not consider effects of aging, biochemical factors, genetic factors, posture, muscular status or regular exercise, which may have a role in the development of knee osteoarthritis^40^. Some of these may be taken into account in the future if known, such as age (through tissue failure limit^26^) and physical activities (through cumulative stress levels). However, it should be remembered that this was the first attempt to simulate cartilage degeneration of subjects complemented by clinical findings, and implementation of other than mechanical factors into the model would necessitate first an extensive set of *in vitro* validation data.

Physical activity levels can be measured with either wearable devices or with physical activity scales for the elderly (PASE)^37^,^41^. However, it was recently discussed that the physical activity level itself may not be the reason for the onset of osteoarthritis, but it was rather suggested that knee loads during per-unit-distance (PUD) may explain the risk for the onset of osteoarthritis. For instance, it was presented that PUD loads are identical between running and walking, whereas PUD knee adduction moments (KAM) are higher during walking^35^. This may be one of the reasons why runners don’t have an increased risk for the onset of knee osteoarthritis. In other words, the risk of osteoarthritis is not necessarily increased with the increase in local stress levels if those are only short-term stresses (not cumulatively accumulated stresses in a local area). Our degeneration algorithm is currently able to simulate cumulative sum of local stresses as a function of time and it produces similar PUD load values between walking and running as in the aforementioned study^35^. Since moderate physical activity is much more common than high intensity activity, particularly for overweight people, we believe that our approach to focus only on forces produced by walking is sufficient. Nevertheless, it should be highlighted that low level activities, especially prolonged kneeling/squatting, are also risk factors for the onset of knee osteoarthritis^15^. However, such loadings are typically related to occupational hazard, and validity of implementation of those kind of loadings into the algorithm would be a great challenge. Inclusion of different daily activities would increase substantially the complexity of the model, but should be considered in the future for fully subject-specific evaluation.

Though the onset and progression of osteoarthritis are separate processes, our degeneration algorithm does not separate these two processes. However, differentiation of the onset and progression of osteoarthritis is not trivial. For instance if there is overloading of cartilage causing the onset of osteoarthritis, it may be followed by several factors, such as inflammation, which then obviously can alter criterion for the progression of osteoarthritis. On the other hand, clear damage to the tissue may mean that the areas surrounding the damage may have lowered susceptibility to external forces. However, the algorithm was developed with different linear and nonlinear criteria for the onset and development of osteoarthritis. When comparing our results with experimental Kellgren-Lawrence grades, the nonlinear approach (equation 1) with a rapid development of collagen fibril degeneration (from Kellgren-Lawrence grade 0 to higher grades) followed by slower progression of osteoarthritis (from Kellgren-Lawrence grade 1 or 2 to higher grades) showed the best match with experiments (Fig. 6b) and was consistent with an earlier study^27^.

In order to thoroughly validate the algorithm, more subjects from different groups should be modeled and compared to experimentally observed osteoarthritis grades before the algorithm will be ready for clinical use. The algorithm should also be validated against experimentally determined levels of osteoarthritis simultaneously for different compartments (patellofemoral, medial and lateral tibiofemoral). These are our future goals, as well as accounting for that the tensile failure limit for the collagen fibril degeneration may vary due to several factors, such as gender, age and site (femur and tibia)^26^,^27^. However, it should be noted that the algorithm presented here worked well with one single threshold value for two relevant subject groups, indicating the potential of the algorithm.

In the future, the presented approach could be applied as a clinical tool to simulate the development of knee osteoarthritis of a subject with a sport injury, joint disorder, overweight, and help to evaluate the best possible intervention. This algorithm would help an orthopedist to make subject-specific predictions for the development of knee osteoarthritis, and estimate the effect of interventions (*e.g.,* osteotomy, meniscectomy, meniscus replacement, weight loss) on the future cartilage health before they are applied to the patient. Furthermore, the presented approach could even be applied to optimizing surgical operations (*e.g.*, size of meniscus removal, properties of ligament reconstruction) and therefore, possible surgical re-operations could be avoided more efficiently and costs due to re-operations would be minimized. However, the current algorithm was tested here only for two subject groups (normal weight and overweight). Thus, in order to achieve these future goals, the current method needs to take into account more subject-specific features and processes occurring in osteoarthritis (other than collagen network degeneration), and it needs more validation data from various subject groups.

## Methods

### Physical activity and cartilage degeneration

It has been shown that loads applied at a high strain rate are one of the most crucial risk factors for the onset and development of osteoarthritis^36^,^42^,^43^,^44^. Physical activity levels can be categorized into 3 different intensity levels (low, moderate, high). The daily percentage fractions for low, moderate and high intensity physical activity levels have been suggested to be ~65%, ~30% and ~5%, respectively^45^. Since low intensity physical activities are mainly composed of lying, sitting or standing, the joint forces generated by the low activity level are not crucial to take into account in our cartilage degeneration algorithm which is based on mechanical overloading. Moderate intensity physical activity, such as walking, is ~six times more frequent than high intensity activities (sport) and this relationship is naturally expected to increase with increased age and weight. In the current study, the mean age of the test subjects was over 50 years. Furthermore, the first signs of osteoarthritis are typically found in older people. Thus, we suggest that moderate physical activities can cause excessive joint forces for overweight patients, which may lead to degenerative changes in cartilage throughout life. Therefore, only joint forces generated by the moderate intensity physical activity (normal walking) are considered in the current algorithm.

### Osteoarthritis Initiative Data Usage

Subjects were chosen based on the following OAI datasets: AllClinical00 and AllClinical06 for BMI, age, previous knee injuries, health status of menisci; kxr_sq_bu00, kxr_sq_bu06 for Kellgren-Lawrence grades. OAI datasets 0.C.2 and 6.C.1 were used for baseline and 4-year follow-up MRI and x-ray (bilateral posterior-anterior projection with knees flexed to 20–30 degrees and feet rotated 10 degrees) data, respectively. Ethical approval for collecting all subject information was provided by the OAI. Knee MRI’s were carried out in accordance with FDA guidelines, whereas knee radiographs (x-ray) were carried out in accordance with typical guidelines for annual and total radiation dosage to research subjects. Written consent was obtained from all subjects prior to each clinic visit. The committee on human research is the institutional review board for the University of California, San Francisco (UCSF), and its affiliates. Since data collection was performed in many places, possible changes in OAI study protocol were reviewed and approved by local institutional review boards. Further details related to the OAI data are available in the OAI web-site (https://oai.epi-ucsf.org).

### Statistical analysis

One-way ANOVA with Tukey’s Post-hoc test was used to compare the BMI values between the subject groups with different Kellgren-Lawrence grades. This same test was performed to compare the experimentally observed Kellgren-Lawrence grades at different follow-up points. Furthermore, Pearson (mean values) and Spearman (all observations) correlations were calculated between experimentally observed development of Kellgren-Lawrence grades and simulated collagen fibril degeneration. SPSS software (v. 21) was used for statistical comparisons (SPSS, Inc., Chicago, IL).

### Model geometries and finite element meshes

The baseline and 4-year follow-up MRI data of the right knee joints were obtained from the OAI database (http://www.oai.ucsf.edu/) using datasets of 0.C.2 and 6.C.1, respectively. The images were obtained with a clinical 3 T MRI system (Siemens Magnetom Avanto, Erlangen, Germany) using a sagittal dual echo steady-state (SAG 3D DESS) imaging sequence (TR = 16.32 ms, TE = 4.71 ms, in-plane resolution = 0.36 mm, slice thickness = 0.7 mm). Then, the MRI data information was imported in Mimics v12.3 (Materialise, Leuven, Belgium), where the cartilage and meniscus tissues were manually segmented using a sagittal view and saved as a surface mesh (STL format). Segmentation was performed for each MRI slice where cartilage or meniscus was visible. Due to variations in tissue intensities between MRI slices, especially in cartilage tissue, the intensity thresholds for cartilage-bone interface were ~45% and ~60% from the peak intensity of cartilage (after subtracting bone) in tibial and femoral cartilage, respectively. Next, the surface meshes were converted to solid geometries (SAT format) using Matlab (R2012b, The Mathworks, Natick, MA, USA). Finally, the solid geometries were imported into Abaqus finite element package (v6.13-3, Dassault Systèmes, Providence, RI, USA), where the finite element meshes for the cartilage and meniscus tissues were constructed. The same investigator who performed segmentations performed also simulations with the knowledge of subject IDs and Kellgren-Lawrence grades.

First-order 8-node porous continuum elements (type = C3D8P) were used for tibial and femoral cartilage, whereas first-order continuum elements without pore pressure (type = C3D8) were used for the menisci. In order to achieve a good contact convergence between master and slave surfaces in the whole knee joint models during the entire gait cycle loading, the femoral cartilage was adjusted as a master surface for the menisci and the tibial cartilage, while the menisci were adjusted as a master for the tibial cartilage. Element sizes of ~1.0 mm, ~0.7 mm and ~1.5 mm with 3 depth-wise element layers were adjusted for the menisci, tibial and femoral cartilage meshes, respectively. Furthermore, to improve convergence in the contact between the femoral cartilage and meniscus, the superficial element layers in the menisci were halved with a subdivide option. In the compartment models, which were used with the collagen fibril degeneration algorithm, the element sizes of 0.5 mm and 1.0 mm were adjusted for the tibial and femoral cartilage. High mesh densities between the femoral (master surface) and tibial cartilage (slave surface) were used in order to ensure model convergence even with high tissue deformation between the master and slave surfaces caused by severe local collagen fibril degenerations.

### Contact definitions

At each contact, surface-to-surface contact discretization and finite sliding tracking with hard contact option for pressure-overclosure relationship with Abaqus default contact enforcement (penalty) methods were used. Tangential behavior was assumed to be frictionless using Abqus default (penalty) friction formulation by using zero as the friction coefficient. Master surfaces were defined as surfaces, whereas slave surfaces were defined as nodes. Due to dynamic loading, fluid flow can be assumed negligible^46^. Therefore, free fluid flows were restricted through cartilage surfaces (Abaqus default).

### Boundary conditions

The bottom surface of the medial and lateral tibial cartilage was fixed, whereas the bottom surface of the femoral cartilage (the interface between bone and cartilage) was constrained with a coupling constraint option to a reference point located at the middle-central point between the medial and lateral epicondyles of the femur (Fig. 3)^47^. This enabled us to control the femoral motion with respect to the tibia by the changes in boundary (or loading) conditions at the reference point (see below). Meniscal movements were restricted by using linear spring elements, mimicking the meniscal attachment ligaments. The end nodes of each meniscal horn were fixed to anchorage points of meniscal attachments, and the stiffness of each attachment (sum of all spring constants at each horn) was 350 N/mm^48^.

### Implementing gait load

Since subject-specific gait data was not available, the experimental gait cycle was obtained from earlier experimental studies (Fig. 3). The experimental gait data was first quantified with 20 equal spaced time intervals between 0 – 1 s (stance phase) using 12^th^ order *polyfit* function in Matlab. Then, similarly as in the earlier study^47^, the gait cycle input was implemented into the reference point, located at the middle-central point between the medial and lateral epicondyles of the femur (Fig. 3), using a time dependent boundary condition with a tabular option in Abaqus. Before the gait load was introduced, initial loads must be generated within the cartilage-cartilage and the cartilage-meniscus interfaces. This was done by applying small axial translation of the reference point (until initial cartilage-meniscus-cartilage contact was achieved), which was followed by the initial axial load (the initial axial load from the experiments). The gait was finally controlled by the varying extension-flexion rotations and axial loadings (scaled to the subject’s bodyweight), while varus-valgus was allowed to rotate freely according to the contact surfaces of the femur and tibia, similarly as was done earlier^47^. Due to the small variations in the anterior-posterior and medial-lateral translations and non-systematic internal-external rotation pattern between different subjects^49^,^50^, these motions were kept fixed in the current models. Together, these assumptions led to a nearly constant ~50–50% force distribution between the lateral and medial compartments in both knee joint models during the gait load, similarly as presented in previous studies^51^,^52^.

### Material properties for cartilage and meniscus

Cartilage tissues were considered as a fibril reinforced poroviscoelastic (FRPVE) material^53^,^54^, in which the total stress (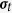) is composed of the sum of the effective solid stress of the non-fibrillar matrix (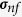), the fibril network stress (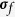, see Fig. 3b), and the fluid pressure 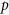 as follows:


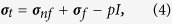


where 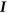 is the unity tensor. The non-fibrillar matrix, describing mainly the proteoglycan matrix, was modeled with a Neo-Hookean porohyperelastic material with the nonfibrillar modulus (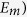, Poisson’s ratio (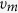) and permeability (*k*). The fibrillar network, describing the collagen network, was modeled to be viscoelastic with the initial fibril network modulus (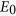), the strain-dependent fibril network modulus (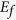) and the viscoelastic damping coefficient (*η)*. The fluid fraction *n*_*f*_ was assumed to be identical in both femoral and tibial cartilages (Table 1). Since the theoretical background of the FRPVE material is completely described in earlier studies^53^,^54^, we focus on justifying the chosen material parameters presented in Table 1. The FRPVE material parameters for the tibial and femoral cartilage were obtained from an experimental study^55^. With those parameters, cartilage deformations in a knee joint model followed nicely those obtained from *in vivo* cartilage deformation experiments of the same subject’s knee using a clinical cone beam CT^46^.

Meniscal tissues were modeled with a transverse isotropic (TI) material behavior with the radial (*E*_*1*_), axial (*E*_*2*_) and circumferential (*E*_*3*_) Young’s moduli, the Poisson’s ratios (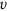
_*12*_and 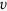
_*13*_) and the shear modulus (*G*_*13*_). These parameters were based on earlier studies^56^,^57^ and they are presented in Table 1. The effect of interstitial fluid was not considered in the meniscus, since only dynamic supportive role of the medial meniscus needed to be taken into account (not the time-dependent behavior).

### Collagen fibril architecture

The collagen fibril network in the FRPVE material was composed of 4 primary and 13 secondary collagen fibrils, which are located at each integration points in each element^53^. While all secondary fibrils are oriented randomly, each primary fibril has a certain orientation, which is dependent on their location within the cartilage tissue. Depth dependent primary collagen fibril architecture was considered in both the tibial and femoral cartilage. In the superficial, middle and deep element layers, primary collagen fibrils were oriented parallel, in a 45 degree angle and perpendicular, respectively, to the contact surfaces. Furthermore, the superficial split-line pattern of femoral and tibial cartilage, based on literature^58^,^59^, was considered as presented in our previous study^60^. In the degeneration algorithm, fibril orientations were not physically altered, but osteoarthritic changes were accounted for in the fibril network stress/stiffness (Fig. 3b) (which is strongly controlled by the orientation (Fig. 2, right)).

### Compartment model for the degeneration algorithm

Experimental observations showed that osteoarthritis developed in the medial joint compartment in the subject group 2 (obese). Thus, the cartilage degeneration algorithm was implemented into the medial compartment in both group 1 and 2 models. Knee joint motion was implemented into the model in two steps. First, the joint rotations (flexion/extension and varus/valgus) and the total medial compartment axial forces were extracted from the simulation outcomes of the whole knee joint models (Fig. 3a). Then, in order to reduce the amount of contacting surfaces (improving model convergence and computational efficiency), the supportive effect of the medial meniscus (calculated as a function of time) was reduced from the extracted axial load, leaving the effect of meniscus to distribute and absorb loads still to the model. Next, the extracted subject-specific rotations and tibiofemoral axial loads of the medial compartment as obtained from the whole joint models (Fig. 3a) were implemented into the compartment model (Fig. 3b). Finally, the iterative cartilage degeneration algorithm was included and the computational models simulated 100 iterative adaptive steps.

### Testing different collagen fibril degeneration algorithm approaches

Since experimental data of the collagen fibril degeneration under excessive loads is not available, we tested various different linear and nonlinear approaches to simulate collagen fibril degeneration. Each of them produced similar final results. Only the rate of degeneration differed. When using linear approaches, the rate of local fibril degeneration was increased with a constant ramp based on the exceeded level of local stress 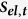 as follows:


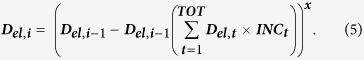


The slope of ramp was controlled by the 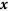 term. When using nonlinear approaches, different indexes for the root term (from 1 to 4) were tested. Based on our testing, we decided to use a nonlinear method (equation 1), in which the collagen fibril degeneration was faster (in arbitrary units) compared to other tested approaches.

**Figure 7:**
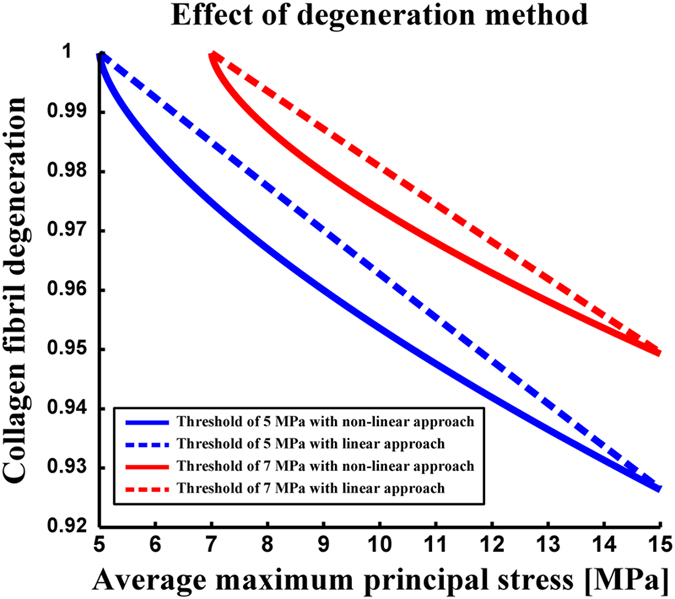
Effect of the degeneration method (linear or nonlinear, equation 1 and equation 5) on the collagen fibril degeneration after one iterative step (one stance phase) in one element with thresholds of 5 MPa (blue curves) and 7 MPa (red curves) for the collagen fibril degeneration. The non-linear method (equation 1) is indicated with solid lines, whereas the linear method is indicated with dashed lines (equation 5 with ***x*** = 4). Non-linear curves represent the collagen fibril degeneration used in the current study. Methods are matched to obtain the same rate of collagen fibril degeneration at 15 MPa stress.

In the linear approaches (equation 5), the degeneration rate was relatively slow, especially within average failure stress values (averaged over one element during one stance phase, *i.e.* one time increment) slightly over the chosen threshold limits for the collagen fibril degeneration (Fig. 7). The non-linear approach is able to produce noticeable fibril degeneration with wider range, especially with stress values between 0 and 1 MPa above the chosen threshold limit for the collagen fibril degeneration (Fig. 7). It should be noted that validation data from the collagen fibril network stiffnesses during progression of osteoarthritis is not available. However, the presented approach produces excellent agreement between experimental measurements (MRI and Kellgren-Lawrence grading) and modeled fibril degeneration (Fig. 6).

## Additional Information

**How to cite this article**: Mononen, M. E. *et al.* A Novel Method to Simulate the Progression of Collagen Degeneration of Cartilage in the Knee: Data from the Osteoarthritis Initiative. *Sci. Rep.* **6**, 21415; doi: 10.1038/srep21415 (2016).

## Figures and Tables

**Table 1:** Implemented material parameters for the fibril reinforced poroviscoelastic (FRPVE) and transverse isotropic (TI) materials.

FRPVE material parameters*	Femoralcartilage	Tibialcartilage
*E*_*m*_ (MPa)	0.215	0.106
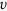 _*m*_	0.15	0.15
*E*_*0*_ (MPa)	0.92	0.18
*E*_*f*_ (MPa)	150	23.06
*η* (MPa s)	1062	1062
*k* (10^−15^ m^4^/Ns)	6	18
*n*_f_**	0.8–0.15z	0.8–0.15
TI material parameters***	**Meniscus**	
*E*_*1*_* *=* E*_*2*_ (MPa)	20	
*E*_*3*_ (MPa)	140	
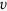 _*12*_	0.3	
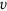 _*13*_	0.2	
*G*_*13*_ (MPa)	57.7	

^*^Obtained from Halonen *et al.* (2014).

^**^Fluid distribution is from surface till bone-cartilage interface, where z indicates normalized depth (surface = 0, cartilage-bone interface = 1).

^***^Obtained from Vaziri *et al.* (2008) & Zielinska *et al.* (2006).
